# Simulating Crystal Structure, Acidity, Proton Distribution, and IR Spectra of Acid Zeolite HSAPO-34: A High Accuracy Study

**DOI:** 10.3390/molecules28248087

**Published:** 2023-12-14

**Authors:** Xiaofang Chen, Tie Yu

**Affiliations:** Institute of Molecular Sciences and Engineering, Institute of Frontier and Interdisciplinary Science, Shandong University, Qingdao 266237, China; yutie@sdu.edu.cn

**Keywords:** zeolite, aluminophosphates, crystal structure, acid, stability, distribution, density functional theory, SCAN meta-GGA approximation, Birch–Murnaghan equation, neutron diffraction

## Abstract

It is a challenge to characterize the acid properties of microporous materials in either experiments or theory. This study presents the crystal structure, acid site, acid strength, proton siting, and IR spectra of HSAPO-34 from the SCAN + rVV10 method. The results indicate: the crystal structures of various acid sites of HSAPO-34 deviate from the space group of $R\bar{3}$; the acid strength inferred from the DPE value likely decreases with the proton binding sites at O(2), O(4), O(1),and O(3), contrary to the stability order in view of the internal energy; the calculated ensemble-averaged DPE is about 1525 kJ/mol at 673.15 K; and the proton siting and the proton distribution are distinctly influenced by the temperature: at low temperatures, the proton is predominantly located at O(3), while it prefers O(2) at high temperatures, and the proton at O(4) assumedly has the least distribution at 273.15–773.15 K. In line with the neutron diffraction experiment, a correction factor of 0.979 is needed to correct for the calculated hydroxyl stretching vibration (ν(O-H)) of HSAPO-34. It seems that the SCAN meta-GGA method, compensating for some drawbacks of the GGA method, could provide satisfying results regarding the acid properties of HSAPO-34.

## 1. Introduction

Acidic silicon-doped silicoaluminophosphates (abbreviated as SAPOs) are developed from the famous microporous material silicoaluminophosphates (AlPOs), produced through the SII and SIII mechanisms, where some P atoms are substituted by the association of Si and H atoms, and P and Al atoms are substituted by two Si atoms, respectively [1,2,3,4,5]. Due to the excellent performance of the catalysis and the adsorption, SAPOs have widespread applications, especially in the field of methanol conversion into olefins (MTO), CO_2_ capture and conversion, NH_3_-SCR, and the DeNO_x_ reaction [5,6,7,8,9,10,11,12,13,14,15,16].

The performance of zeolite is generally determined by the acid site, the acid amount, the acid strength, and accessibility to the acid site [17,18,19,20,21]. Most SAPOs are structurally constructed by isomorphous Si^4+^ displacing P^5+^, and the formed negative charge is effectively compensated for by the proton bound to the framework oxygen or metal cation [4,22] in the channels/cages. It was found that the P sites in SAPOs were substituted exclusively by Si sites [3]. The acid zeolite HSAPO-34 has the acidic center of SiO(H)Al. The compensatory proton is usually regarded as the intrinsic origin of the Brönsted acid of HSAPO-34. The incorporated Si atom directly changes the electrostatic potential of the adjacent O atoms, and then makes the O atom unequivalent. Thus, the acid properties of HSAPO-34 are largely determined by the nature of the incorporated Si species [23]. It seems feasible to identify the Brönsted acid site by examining the chemical environment of the oxygen atom chemically bound to the incorporated Si atom. The acidic single silicon-substituted SAPO-34 zeolite (HSAPO-34) has four unequivalent O atoms (i.e., O(1)-O(4)) chemically bound to the incorporated Si atom. It is expected that HSAPO-34 will have four possible Brönsted acid sites. The acid properties of HSAPO-34 are also influenced by the concentration of Si islands and the proton distribution [23,24].

Some acid properties of HSAPO-34 were studied by experimental technologies, such as neutron diffraction [2,3,25], probe molecule (for instance, NH_3_, CO, and C_2_H_4_) adsorb–desorption [4,11,19,26], IR [26,27] and NMR measurements [23,28], and transmission electron microscopes (TEMs) [11]. An isomorph of HSAPO-34, a chabazite specimen, was found to have the trigonal unit cell with the space group of $R\bar{3}m$ (or nearly so) and alattice parameter of a = 9.40 Å and 94.30° [25]. The infraredmass spectrometry/temperature-programmed desorption (IRMS-TPD) of ammonia indicated that HSAPO-34 had a slightly weaker acid strength than chabazite, and the primary Brönsted OH was on the edge of the Si domain (island) [4]. Among a series of hierarchical SAPO-34 samples, the nanostructure of the SAPO-34 sample with a lower acid concentration and weaker acid strength (i.e., a sample with a lower intensity in the NH_3_-TPD profiles) exhibits the appropriate catalysis performance for MTO reactions [11]. Two distinct peaks were identified as the signal of two bridging hydroxyl Brönsted sites of the crystalline HSAPO-34 catalyst [3,23], and the proton at O(4) likely had a greater acidity than that at O(2) [3]. It was assumed that the protons preferred to reside on the framework O(2) and O(4) atoms in the dehydrated [3] SAPO-34 sample, while the O(1) atom was coordinated by a hydroxonium ion (H_3_O^+^), and yet, the hydrogen bond formed between O(2) and a water molecule in the hydrated sample [2]. Meanwhile, three distinct acid sites of HSAPO-34 were further evidenced by FTIR spectra at 3631, 3617, and 3600 cm^−1^ [26]. The Si mole fraction and the particle size almost had no influence on the acid strength of HSAPO-34 [29], and the metal content had a strong influence on the concentration of acid sites [30].

The acid zeolite HSAPO-34 sample is usually synthesized under hydrothermal conditions. The high synthesis reactivity makes it difficult to monitor the crystal nucleus formation [31,32,33]. The proton exchanges also proceed fast between different adsorption sites [28]. This leads to the HSAPO-34 sample in the experiment being a mixture of various Brönsted acid sites. HSAPO-34 belongs to the small-pore materials, usually with the bore diameter of about 3–12 Å [5]. Some acid sites on the zeolitic surface are easily accessible, while the others, especially in the narrow channel or the inner cavity, are difficult to reach. The fast proton exchange, as well as the inaccessibility, also increases the challenge of characterizing the precise acid properties of SAPOs in the experiment.

The acid zeolite HSAPO-34 has also been investigated by diverse theories. The plane wave periodic gradient-corrected density functional theory (PDFT) is the favorite of researchers to simulate the structure, property, and reactivity of the bulk [34]. The general gradient approximation (GGA) PW91 functional was once used to calculate the exchange correlation energy; O(1) and O(3) were suggested to be two favored proton binding sites of HSAPO-34 [35,36]. In comparison with neutron diffraction results [2,3,28], it has been concluded that the GGA PW91 functional was capable of predicting the lattice parameter and the ion position at a certain level of precision, yet it failed to fully reproduce the acid properties of HSAPO-34 [36]. The divergences between the experiment and the theory are probably caused by the theoretical model’s simplicity, the experimental complexity, and the low energy difference between various Brönsted acid sites of HSAPO-34.

Besides the gradient-corrected correlation PW91 functional [37], PBE is another popular GGA functional [38]. As a simplification of PW91, PBE is capable of yielding numerical results almost identical to PW91 in most usual systems. The nonequivalence of PBE and PW91 has also been observed in the presence of surface effects; and PBE is generally superior to PW91 in treating these weak interactions [39]. During a plane wave theory simulation, the hierarchy of DFT approximations is adopted to resolve the Kohn–Sham equation through the displacement of the genuine potential by the relatively flexible pseudo potential [40,41,42,43,44,45]. The generalized gradient approximation is the second rung of Jacob’s ladder [46], which treats the density at a given point changeably [34]. It is desirable to use the suitable pseudo potential to maximally reproduce the crystal structure and the energy of the real bulk [47]. To compensate for the lack of kinetic energy, the meta generalized gradient approximation (meta-GGA) appears to be another wave plane approximation higher than GGA [48,49,50,51]. Recently, it was reported that the strongly constrained and appropriately normed (SCAN) approximation had the ability to evaluate solid stability and reactivity, although it demanded the long computation time and expensive calculation resources [48,49,50,51,52].

In this study, we attempt to use a higher-level approximation method (i.e., SCAN + rVV10) to simulate the fine crystal structure of all possible Brönsted acid sites of HSAPO-34 and analyze the acidity strength and the proton siting. The preferred proton binding site will be further analyzed in terms of the quantitative proton distribution of HSAPO-34. This study will shed light on the difficulties of accurately simulating the acid properties of porous materials.

## 2. Results and Discussion

### 2.1. Unit Cell

**Figure 1:**
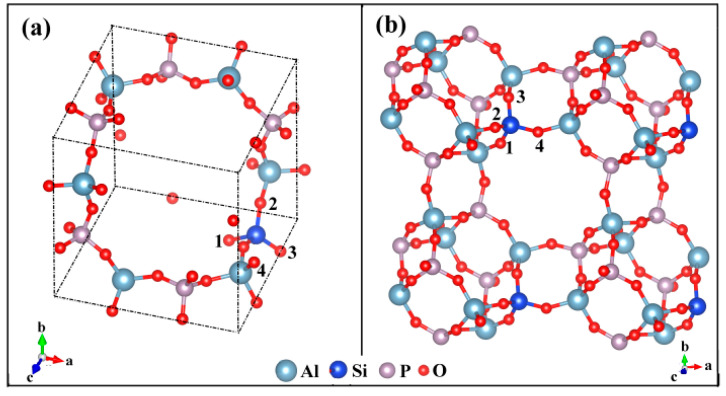
Single silicon-doped alumino-phosphates (HSAPO-34): (**a**) the single-unit cell (1UC) with rhombohedral representation, (**b**) the expanded lattice structure. Note that the proton is omitted for simplicity.

AlPO_4_-34 is structurally analogous to an isomorph, a chabazite. Both have CHA topology and yet have different element compositions. Following the SMIII mechanism, the all-silica chabazite is converted to AlPO_4_-34 by the alternative displacement of Si by Al and P atoms. The space group reduces from $R\bar{3}m$ in the all-silica CHA framework to $R\bar{3}$ in the AlPO_4_-34 framework. It was found that the silicon atom in SAPOs was substituted exclusively by P sites and the formed negative charge was compensated for by the proton, leading to the acid zeolite HSAPO-34 [3]. Chabazite has the primary building units (PBU) of [Si-O-Al] and [Si-O-Si] linkages, while SAPO-34 has [Si-O-Al], [Si-O-Si], and [Al-O-P] linkages. The rhombohedral representation of HSAPO-34 in a single-unit cell (1UC) has the chemical formula of HAl_6_P_5_Si_1_O_24_ (Figure 1). Some atoms in HSAPO-34 are structurally equivalent. The tetrahedral units of AlO_4_ and PO_4_ are coupled in HSAPO-34 crystalline. The Al-O-P bonds are formed and the [O-Al-O-P]_n_ chemical network chains appear in the crystalline of HSAPO-34. The acid zeolite HSAPO-34 has a narrow eight-member ring (8MR) (3.8 Å × 3.8 Å) perpendicular to the crystalline *ac* or *bc* plane, and at welve-member ring (12MR) along the side face of the eight-member ring [53].

Apart from the newly added H atom, HSAPO-34 has a crystal structure similar to the AlPO_4_-34 framework. There are four unequivalent O atoms around the incorporated Si atom, denoted as O(x) (x = 1–4) (Figure 1). Any of the oxygen atoms which are chemically bound to the zeolitic substituted tetrahedral (T) sites could serve as the attached site of the Brönsted acid proton [54]. Each unequivalent O atom is shared by three windows: O(1), two 4MRs and one 8MR; O(2), one 8MR, one 6MR, and one 4MR; O(3), two 4MRs and one 6MR; O(4), two 8MRs and one 4MR. The O atom numbering in this study is the same as that mentioned by Smith et al. [3] and Jeanvoine et al. [36], which differs from the database of zeolite structures in the internal zeolite associate (IZA) [53] (Table 1). The four unequivalent O atoms are around the incorporated Si atom, resulting in four possible Brönsted acid sites of HSAPO-34, denoted as HSAPO-34-O(x), x = 1–4.

### 2.2. Calculated Crystal Structure

Generally, the Birch–Murnaghan equation [55] describes the total energy (E(V)) of the bulk dependence of the volume (V), the equilibrium volume (V_0_), the equilibrium electronic energy (E_0_), the bulk modulus (B_0_), and its derivative with respect to pressure $\left(B_{0}^{\prime }\right)$. On the basis of the E-V data, the total energy and the crystal volume in the equilibrium state could be deduced by fitting the E-V curve with the third order Birch–Murnaghan equation (Equation (1)).

(1) $$E\left(V\right)=E_{0}+\frac{9V_{0}B_{0}}{16}\left\{{\left({\left(\frac{V_{0}}{V}\right)}^{\frac{2}{3}}-1\right)}^{3}B_{0}^{\prime }+{\left({\left(\frac{V_{0}}{V}\right)}^{\frac{2}{3}}-1\right)}^{2}\left(6-4{\left(\frac{V_{0}}{V}\right)}^{\frac{2}{3}}\right)\right\}$$

**Figure 2:**
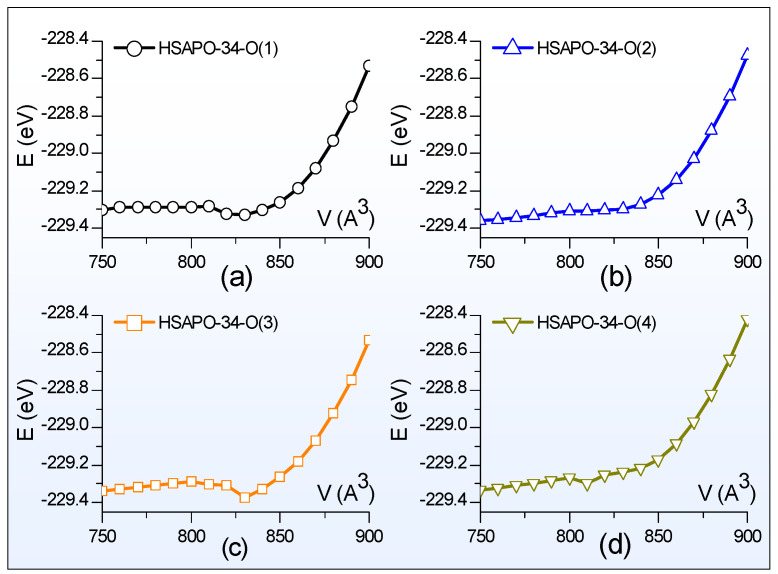
The total energy depends on the lattice volume. The proton binding sites of HSAPO-34 are (**a**) O(1), (**b**) O(2), (**c**) O(3), and (**d**) O(4).

Scanning of the E-V curve is performed to search for the crystal structure in the equilibrium state. Figure 2 illustrates the total energy dependence of the lattice volume for four possible Brönsted acid sites of HSAPO-34. The constant volume selected is from 750 Å^3^ to 900 Å^3^ with an interval of 10 Å^3^. Thus, each E-V curve scan needs 16 structures at a constant volume, which are used to partially optimize. The partial optimization means that the crystal structure is optimized by relaxing the cell shape and the ion position but fixing the cell volume. The atomic force and the stress tensor are calculated. As shown in Figure 2, each curve corresponds to one Brönsted acid site of HSAPO-34, and all exhibit the parabola shape. The total energy of each curve at 750 Å^3^ is always lower than that at 900 Å^3^.

The fitted parameters of E_0_, V_0_, B_0_, and $B_{0}^{\prime }$ for each Brönsted acid site of HSAPO-34 are provided in Table 2. When the proton is chemically bound to the O(1) or O(3) atom of the acidic zeolite HSAPO-34, the equilibrium volume (V_0_) of the Brönsted acid site is equal to 821.82 Å^3^ or 820.13 Å^3^. Previous neutron diffraction experiments detected that the lattice volume of HSAPO-34 was 822.24 Å^3^ for the dehydrated sample [3] and 820.20 Å^3^ for the hydrated sample [2]. In comparison, two Brönsted acid sites, HSAPO-34-O(1) and HSAPO-34-O(3), have lattice volumes that are the most close to the experimental value. When the proton rests on the O(2) or O(4) atom, the corresponding Brönsted acid site has a negative lattice volume deviation from either the dehydrated HSAPO-34 (−11.26 and −29.06 Å^3^) [3] or hydrated sample (−9.22 and −27.02 Å^3^) [2]. It is worthwhile noting that the lattice volume detected by the neutron diffraction experiment is generally related to the Brönsted acid site mixture of the acid zeolite HSAPO-34 in the equilibrium state.

To obtain the more precise crystal structure of HSAPO-34, full optimization is carried out by the period DFT, implemented with the strongly constrained and appropriately normed (SCAN) method. The atomic force and the stress tensor are calculated by relaxing the volume and the ion. Table 3 lists the calculated lattice parameters for four Brönsted acid sites of the acidic zeolite HSAPO-34. The dehydrated HSAPO-34 [3] is selected as the reference. For the detailed atomic coordinates of the calculated crystal structure of HSAPO-34, please refer to Supplementary Materials.

As shown in Table 3, three crystal edges (i.e., a, b and c) from each Brönsted acid site of HSAPO-34 are not completely equal to each other. Taking HSAPO-34-O(1) as an example, the crystal edge of a is calculated to be 9.40 Å, equal to the deduced experimental value of dehydrated HSAPO-34 [3], while the crystal edge of b increases by 0.09 Å and the crystal edge of c decreases by 0.14 Å. The crystal angle of α of HSAPO-34–O(1) is the calculated value the most close to the deduced experimental value. Among four Brönsted acid sites, HSAPO-34-O(1) has the least absolute values of both mean absolute deviation (MAD) and root mean square deviation (RMSD), followed by HSAPO-34-O(2). In total, the absolute value of MAD-abc is within 0.08 Å and the RMSD-abc ranges from 0.02 to 0.14. This implies that each Brönsted acid site of HSAPO-34 has already deviated from the space group of $R\bar{3}$.

As for the lattice volume, one of the Brönsted acid sites with the proton at O(2) is the most close to the dehydrated HSAPO-34 sample reported by the neutron diffraction experiment [3], followed by the Brönsted acid site with the proton at O(1). Relative to the dehydrated HSAPO-34 sample, the absolute values of the lattice volume deviations are within 2.89 Å^3^ when the proton is located at O(1) or O(2), while it rises to 27.13 Å^3^ or 14.43 Å^3^ when the proton is chemically bound to O(4) or O(3).

### 2.3. Thermal Stability, Acidity Strength, and Vibrational Frequency of Brönsted Hydroxyl (ν(O-H))

#### 2.3.1. Thermal Stability

The calculations indicated that four possible acid sites of HSAPO-34 had different internal energy (E), as shown in Table 4. Among them, the acid sites of HSAPO-34 with the proton at O(3) are at the bottom. When the proton switches to be chemically bound to the framework O(1), O(4), or O(2) atom, the corresponding Brönsted acid site’s internal energy increases by 1.025, 1.590, or 1.935 kcal/mol, respectively. Table 4 shows that the proton at O(2) has a higher internal energy than the others. This is probably because the proton at O(2) is lying at an intersection between two 8MR windows. The proton at O(3) has the lowest internal energy and can be regarded as the most stable acid site considering internal energy. The proton at O(3) is almost located in the plane of 6MR; as calculated, it is about 2.703, 2.838, 3.568, 2.572, and 2.864 Å away from the other five O atoms of 6MR. The hydrogen bond likely forms between the proton H atom and the other five O atoms of 6MR, respectively. This probably accounts for the proton at O(3) being the most stable acid site considering internal energy.

Generally, the lower the internal energy, the higher the stability is. The relatively stability of the Brönsted acid site of HSAPO-34 might increase in order of the proton binding sites of O(2), O(4), O(1), and O(3) when the temperature influence is not considered. Previous neutron diffraction experiments discovered that protons preferred to reside on the framework O(2) or O(4) atom in the dehydrated SAPO-34 sample [3], while the O(1) atom was coordinated by a hydroxonium ion (H_3_O^+^), and yet the hydrogen bond formed between O(2) and a water molecule in the hydrated [2] sample. Based on those neutron diffraction experiments, Jeanvoine et al. concluded that the thermal stability of HSAPO-34 increased in order of the proton binding sites of O(2), O(4), and O(1) [2,3]. In view of the relative internal energy, the stability order of various acid sites of HSAPO-34, predicted by the SCAN + rVV10 method, is almost fully confirmed by the experiment [2,3,28].

#### 2.3.2. Vibrational Frequency of Brönsted Hydroxyl (ν(O-H))

The stretching frequency of Brönsted hydroxyl (ν(O-H)) of the acid zeolite HSAPO-34 has received wide attention [3,26,27,35,36,56,57,58]. Previously, Zubkov et al. [27] and Martins et al. [26] experimentally detected that the peaks at 3625/3631 and 3600 cm^−1^ were assigned to the fundamental stretching vibrations of two types of bridged hydroxyl groups at O(1) and O(2) in crystalline SAPO-34, respectively. Martins et al. [26] also detected a third distinct peak at 3617 cm^−1^. Yet, Smith et al. [3,56] used neutron diffraction technology to suggest that the ν(O-H) was 3625, 3601, and 3630 cm^−1^ for HSAPO-34 with protons at O(1), O(2), and O(4), respectively. On the basis of the fully optimized geometries in Section 2.2, phonon calculations of four acid sites of HSAPO-34 were carried out by using the SCAN + rVV10 method, providing some information on the vibrational frequencies. The ν(O-H) of the acid zeolite HSAPO-34 is calculated to be 3706, 3684, 3625, and 3700 cm^−1^ when the proton resides on the framework O(1), O(2), O(3), and O(4) atoms, respectively (Table 4). Our calculated ν(O-H) is very close to the experimental values [3,26,56], with an increment of 25–75 cm^−1^. The ν(O-H) of chabazite, calculated by the PDFT method with the GGA approximation of Perdew and Wang [59], was 3875, 3920, 3820, and 3815 cm^−1^ for the protons at the O(1), O(2), O(3), and O(4) atoms, respectively [58], which are about 100 cm^−1^ larger than our calculation results from HSAPO-34. It is common for almost all GGA functional to overestimate stretching vibrations [60]. In line with the neutron experiment [3,56], an average correction factor of about 0.979 might be introduced to our calculated vibrational frequencies of HSAPO-34 from the SCAN + rVV10 method.

#### 2.3.3. Proton Siting and Proton Distribution

**Figure 3:**
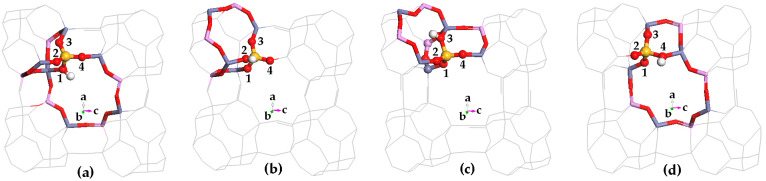
Schematic diagram for four possible Brönsted acid sites of HSAPO-34. The proton binding sites are (**a**) O1, (**b**) O2, (**c**) O3, and (**d**) O4. Noting that Al in cycn, Si in blue, P in purple, O in red, and the number in figure is the numbering of O atom.

Proton siting. Proton siting and proton distribution play crucial roles in the hydrogen bond adsorption and the catalysis reaction [61]. Proton siting would be recognized by examining the calculated crystal structure of the Brönsted acid site [62]. As shown in Table 1 and Figure 3, HSAPO-34 has four types of protons: (1) the proton located at the framework O(1)atom (i.e., H(O1)) is almost at 8MR window, pointing to the positive direction of the crystal axis of b, and the H-O(1) bond is nearly parallel to the crystal plane of [010]; (2) the proton bound to O(2) (i.e., H(O2)) lies at an intersection between two narrow 8MR windows along the [010] and [100] planes, and H(O2) points toward the cavity of the 12-member ring; (3) H(O3) lies at an intersection between the 6MR and 4MR windows, and the H(O3) bond is almost parallel with the [001] plane, and (4) H(O4) is almost at the 8MR windows, and the H(O4) bond is almost parallel with the [100] plane.

Proton distribution. The experiments demonstrate that the proton siting is dependent on the synthesis conditions and is not random [63], but the distribution follows the law of thermodynamics under a given condition [29,64]. Therefore, the possible distribution of four possible Brönsted acid sites of HSAPO-34 could be estimated through Maxwell–Boltzmann statistics [65].

(2) $$<N_{i}>\;=\frac{N{\cdot g}_{i}\cdot e^{-\frac{G_{i}(T)}{k_{b}\cdot T}}}{\sum_{1}^{i}\left(g_{i}\cdot e^{-\frac{G_{i}(T)}{k_{b}\cdot T}}\right)}$$

(3) $$G\left(T\right)=H-T\cdot S$$

(4) $$S=S_{e}+S_{t}+S_{r}+S_{v}$$

(5) $$S={\left(\frac{\partial F}{\partial T}\right)}_{V,N}$$

(6) $$F=-k_{b}\cdot T\cdot lnq^{N}$$

Equation (2) shows the distribution of Maxwell–Boltzmann statistics. The natural abundance (<N_i_>) of the i^th^ configuration is related to the total number of particles (N), the free energy of the i^th^ configuration (G_i_), the temperature (T in Kelvin), and the Planck constant (k_b_). The Gibbs free energy (G(0)) at 0 K is given directly from the VASP calculation results, which are numerically equal to the enthalpy at 0 K. According to statistical mechanics, the Gibbs free energy (G(T)) at a given temperature is associated with the enthalpy (H), the temperature (T), and the entropy (S) (Equation (3)). Herein, the entropy (S) consists of the electric (S_e_), the transitional (S_t_), the vibrational (S_v_), and the rotational (S_r_) contribution (Equation (4)). The entropy stems from the derivative of the Helmholtz free energy (F) divided by the temperature at the constant volume and the constant number of particles (Equation (5)). And the Helmholtz free energy (F) is calculated from the partition function (q) (Equation (6)).

The selected temperature ranges from 273.15 K to 773.15 K with a temperature interval of 50.00 K. The acidic single-silicon substituted SAPO-34 zeolite, HSAPO-34, has four possible Brönsted acid sites. The four calculated Brönsted acid sites are with the space group of P1, and the degeneracy g_i_ in Equation (2) is cancellable to 1. When the numerical values of N, G_i_, g_i_, k_b_, and T are introduced to the Maxwell–Planck statistics equation (Equation (2)), it initiates the distribution of the Brönsted acid site of zeolite HSAPO-34 and further leads to proton distribution.

**Figure 4:**
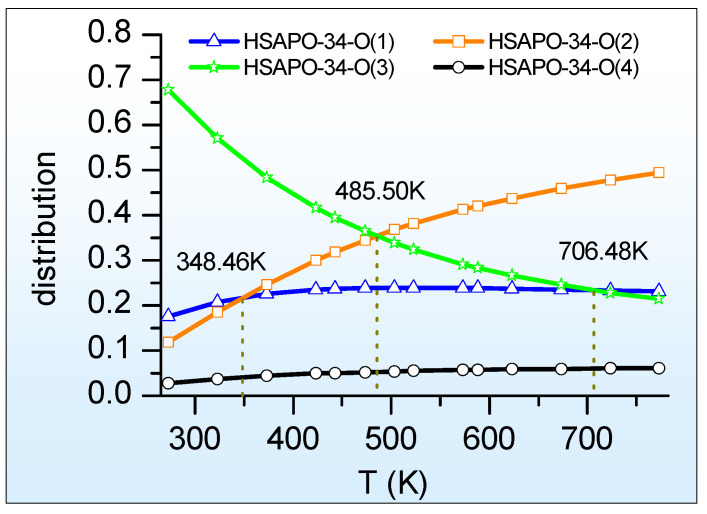
The proton distribution of HSAPO-34 depends on the temperature.

Figure 4 illustrates the relationship between the proton distribution of HSAPO-34 and the temperature, as calculated by the periodic density functional theory with the SCAN + rVV10 approach. As shown, the proton distribution at either O(1) or O(4) varies weakly with the temperature change, while the temperature has a strong influence on the proton distribution at both O(2) and O(3). The HSAPO-34-O(3) distribution gradually declines with an increasing temperature, contrary to the other three Brönsted acid sites of HSAPO-34.

Predominate distribution. At 273.15–485.50 K, the predominant proton is assumedly located at the framework O(3) atom of HSAPO-34, and its contribution is about 67.85–35.28%. The proton at O(3) is almost in the 8MR window along the [100] plane. Searching for the largest proton distribution, the O(2) site competes with the O(3) site heavily. Above about 485.50 K, the proton at O(2) exceeds that at O(3) in abundance. At 485.50–773.15 K, the O(2) site of HSAPO-34 becomes the most dominant proton binding site, contributing to 35.28–49.36% of the proton. As shown in Table 1, the proton located at the framework O(2) atom mainly lies in an intersection of two 8MR windows. The proton siting with the dominant abundance at high temperatures probably contributes the experimental phenomenon that most catalysis reactions mainly take place in the specific 8MR window of HSAPO-34.

Secondary distribution. Figure 4 also shows that the O(1) site of HSAPO-34 competes with two other proton binding sites of O(2) and O(3) heavily. Below 348.46 K, the proton at O(1) is less than that at O(3) in abundance, and then the O(1) site becomes the secondary dominant Brönsted acid site. This is in line with the previous theoretical suggestion that the O(1) and O(3) sites are the two preferred binding sites of the proton [35]. When the temperature ranges from 348.46 K to 706.48 K, the proton at O(1) assumedly accounts for the third largest contribution, occupying 21.67–23.33%. Among four possible Brönsted acid sites, HSAPO-34 with a proton at O(4) assumedly has the least proton distribution, which is below 6.1% at 273.15–773.15K.

In sum, various Brönsted acid sites of HSAPO-34 are calculated to be dependent on the temperature in abundance, resulting in proton siting and its distribution change with the temperature.

#### 2.3.4. Acid Strength

**Figure 5:**
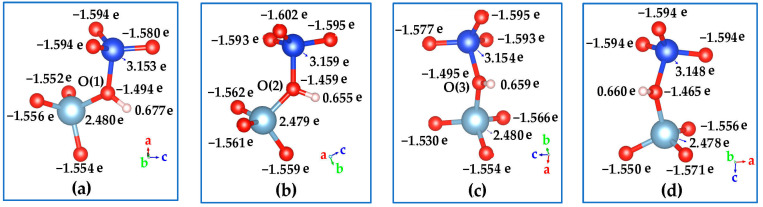
Bader charges on AlO_3_-O(H)-SiO_3_ fragment from four possible Brönsted acid sites of HSAPO-34. The proton binding sites are (**a**) O(1), (**b**) O(2), (**c**) O(3), and (**d**) O(4). Noting that Al in cyan, Si in blue, and O in red.

The charge distribution on the acid zeolites is of considerable interest. Figure 5 shows the calculated Bader charges [66] on the AlO_3_-O(H)-SiO_3_ fragment from four possible Brönsted acid sites of HSAPO-34. The Bader charge analysis is based on the full optimized geometry. All oxygen atoms around the incorporated Si atom appear to have a negative charge (about −1.580 e~−1.602 e), which is slightly lower than those (−1.530 e~−1.566 e) bound to the Al atom. The Bader charge on either the Si or Al atom is less than its respective valence electron; the former ranges from 3.148 e to 3.159 e, while the latter ranges from 2.478 e to 2.480 e. The proton charges from the four Brönsted acid sites are slightly different, calculated to be 0.677 e, 0.655 e, 0.659 e, and 0.660 e when the proton is at O(1), O(2), O(3), and O(4), respectively (Table 4 and Figure 5). In view of proton charge, the acid strength would gradually reduce in order of the proton binding site of O(1), O(4), O(3), and O(2).

DPE = E(H^+^) + E(Z^−^) − E(HZ) (7)

The deprotonated energy (DPE) is capable of reflecting the acid strength of the solid acid catalyst. The deprotonated internal energy is defined as the internal energy difference between the deprotonated (Z^−^) plus the bare proton (H^+^) and the protonated (HZ) zeolites (Equation (7)). It is worth noting that the deprotonated zeolite has a negative charge. For the deprotonated HSAPO-34, its initial structure used for the E-V scan comes from any possible acid sites of HSAPO-34 by eliminating the Brönsted acid proton, and its precise structure would be obtained through a four-step procedure similar to that in Section 2.2. In comparison with the deprotonated HSAPO-34, the Si-O(H) bond length in each acid site of HSAPO-34 is elongated by about 0.134–0.138 Å in the presence of the proton. According to Equation (7), the calculated DPE value is 1526, 1522, 1530, and 1524 kJ/mol when HSAPO-34 is with the proton at O(1), O(2), O(3), and O(4), respectively (Table 4). In general, the less DPE, the stronger the acid strength is. The acid strength assumedly reduces in order of the proton binding site of O(2), O(4), O(1), and O(3). The DPE differences between one other are within 8 kJ/mol, and then those four acid sites have no vast difference in acid strength. Our calculated DPE value for each Brönsted acid site of HSAPO-34 is 20–29 kJ/mol lower than that for the zeolite chabazite previously reported by Jone and Iglesia [67]. It is worth noting that the DPE values are also dependent on the Si island [4].

(8) $$<DPE>=E_{Z^{-}}+E_{H^{+}}-<E_{ZH}>$$

Recently, Jone and Iglesia [67] put forward the ensemble-averaged DPE in view of the statistics (Equation (8)). Herein, <DPE> is the ensemble-averaged DPE value in the thermal equilibration state, $<E_{ZH}>$ is the ensemble-averaged energy of four possible Bronsted acid sites at each atom location of the acid source. It could be estimated by the statistical average over the internal energy and the free energy of each Brönsted acid site (Equation (9)). The item of free energy is related to the probability of the proton.

(9) $$<E_{ZH}>\;=\frac{\sum_{i=1}^{4}\left[E_{ZH,i}\cdot \left(e^{\frac{-G_{ZH,i}(T)}{k_{b}\cdot T}}\right)\right]}{\sum_{i=1}^{4}\left(e^{\frac{-G_{ZH,i}(T)}{k_{b}\cdot T}}\right)}$$

**Figure 6:**
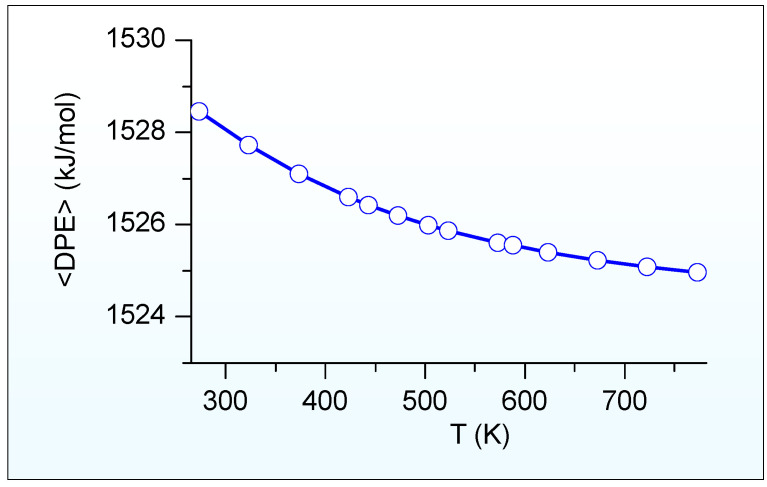
Calculated ensemble-averaged DPE (<DPE>) of HSAPO-34 depends on the temperature.

Figure 6 illustrates the calculated ensemble-averaged DPE (<DPE>) of HSAPO-34 dependent on the temperature. As we predicted, the <DPE> is about 1525 kJ/mol at a suitable methanol-to-olefins catalysis temperature of 673.15 K [6]. The <DPE> value gradually reduces as the temperature increases. The change magnitude of <DPE> is within 3.49 kJ/mol when the temperature ranges from 273.15 K to 773.15 K.

## 3. Materials and Methods

All calculations are performed by the Vienna Ab initio Simulation Package (VASP) [68,69,70,71] using the plane-wave periodic gradient-corrected density functional theory (DFT) methods. The strongly constrained and appropriately normed (SCAN) meta-generalized-gradient approximation (meta-GGA) [48,49,50,51] is used with PAW [72] Perdew–Burke–Ernzerhof (PBE) [38] pseudopotentials (version 52) and the rVV10 nonlocal correlation functional of Peng et al. [51]. This theoretical calculation approach is also called the SCAN + rVV10 method [51], implemented in VASP packages.

The initial structure of HSAPO-34 is constructed through the isomorphous substitution of silicon atom in the all-silica CHA structural topology by following the SMIII mechanism [73]. The CHA structure is obtained from the Database of Zeolite Structures in Internal Zeolite Association (IZA) [53]. The acidic single-silicon substituted SAPO-34 zeolites (HSAPO-34) with rhombohedral representation are selected as the calculation model. The model zeolite HSAPO-34 is the single-unit cell containing six Al atoms, five P atoms, one Si atom, twenty-four O atoms, and one H atom.

The lattice parameter is calculated from the crystal structure of zeolite in the equilibrium state [55]. Prior to the full optimization, scanning of the E-V curve is carried out by partially optimizing a series of crystal structures for each Brönsted acid site at the constant volume. The volume is selected from 750 Å^3^ to 900 Å^3^ with an interval of 10 Å^3^. Fitting the E-V curve with the Birch–Murnaghan equation would produce a rough crystal structure, which is used as the starting crystal structure for full relaxation of the cell shape, the lattice volume, and the ion position. The full optimized structure is further verified as the desirable structure of zeolite HSAPO-34 by phonon analysis. The energy cutoff is set to 570 eV for the E-V scan and 900 eV for the full optimization. The convergence threshold selected is 1 × 10^−2^ eV/Å for the atomic force and 1 × 10^−7^ eV for the energy. The K-point meshes are selected as 2 × 2 × 2 to sample the Brillouin zone for HSAPO-34. The density functional perturbation theory (DFPT) was used to calculate the second derivatives (Hessian matrix and phonon frequencies) without the symmetry restriction. Three small imaginary frequencies (below 6i cm^−1^) of each acid site of HSAPO-34 are present yet not easily removed, which would have an influence on the relative distribution of the proton. An analysis of each imaginary vibration mode indicates that the associated atoms oscillate almost along the same direction. To some extent, the associated nuclear motions are regarded as the translations/rotations of the species. The entropy contribution from 12 cm^−1^ harmonic vibration approximates is almost equal to that from a pseudotranslation/rotational degree of freedom [74,75,76]. In this study, the entropy contribution from those small imaginary frequencies was calculated after they were replaced by 12 cm^−1^ to reduce as much inaccuracy derived from the imaginary frequencies as possible.

The electronic charges on the atoms of each acid site of HSAPO-34 were analyzed by the Bader charge method developed by the Henkelman group [66] on the basis of the full optimized geometry of HSAPO-34.

## 4. Conclusions

Through a four-step procedure, the fine crystal structure of HSAPO-34 has been accurately simulated by the periodic density functional theory with the SCAN + rVV10 method. Four unequivalent framework O atoms around the incorporated Si atom of HSAPO-34 would produce four possible Brönsted acid sites, deviating from the perfect space group of $R\bar{3}$. Under this high-level approximation (i.e., SCAN-meta-GGA), the calculated structural properties on the Brönsted acid sites of HSAPO-34 match the experiment well [2,3,28], including the lattice parameter, the internal ion position, the acid strength, the proton siting, and the relative thermal stability. It seems that the meta-GGA method could compensate for the some drawbacks of the GGA method [36] and then give rise to the satisfying calculation results of the acid zeolite HSAPO-34. The statistics analysis further indicates that both the proton distribution and the ensemble-averaged DPE of HSAPO-34 are distinctly altered by the temperature. It is worth noting that the numerical data mentioned in this study may fluctuate more or less with the selected method, especially on the natural abundance and the temperature threshold.

## Figures and Tables

**Table 1:** The atomic numbering relationship of AlPO_4_-34 framework between this work (same as refs. [3,36]) and the database of zeolite structures in the internal zeolite associates (IZA), as well as the location of O atom.

This work	O(1)	O(2)	O(3)	O(4)
IZA	O(4)	O(3)	O(1)	O(2)
Location	4MR(2) ^α^ 8MR(1,+) ^α,β^	4MR(1) ^α^ 6MR(1) ^α,β^ 8MR(1,+)	4MR(2) ^α^ 6MR(1,+) ^α,β^	4MR(1) ^α^ 8MR(2,+) ^α,β^

^α^ The number in parentheses is the number of window ring; ^β^ The symbol “+” in parentheses is the rough location that the Brönsted acid proton points toward.

**Table 2:** The fitted parametersof the Birch–Murnaghan equation.

Parameters	Brönsted Acid Sites of HSAPO-34			
	O(1)	O(2)	O(3)	O(4)
V_0_/Å^3^	821.82	810.98	820.13	806.62
E_0_/eV	−229.33	−229.33	−229.34	−229.31
B_0_	0.13	0.08	0.12	0.01
$B_{0}^{\prime }$	−27.37	−43.85	−30.35	−400.24

**Table 3:** The calculated lattice parameters of HSAPO-34 with the rhombohedral presentation.

Parameters	Brönsted Acid Sites of HSAPO-34				
	O(1)	O(2)	O(3)	O(4)	Exp [3]
a (Å)	9.40	9.42	9.40	9.19	9.40
b (Å)	9.49	9.39	9.52	9.38	9.40
c (Å)	9.26	9.42	9.32	9.39	9.40
α (°)	94.12	94.79	93.58	95.77	94.27
β (°)	94.13	94.36	94.16	96.12	94.27
γ (°)	94.03	94.67	95.58	96.04	94.27
V_0_ (Å^3^)	819.50	824.46	807.96	795.26	822.39
MAD-abc	−0.02	0.01	−0.05	−0.08	-
RMSD-abc	0.10	0.02	0.14	0.12	-
MAD-αβγ	−0.18	0.34	−0.70	1.71	-
RMSD-αβγ	0.18	0.38	0.85	1.71	-

**Table 4:** The relative internal energy (ΔE), the deprotonated internal energy (DPE), and the stretching frequency of hydroxyl (ν(O-H)) for the acid zeolite HSAPO-34.

Parameters	Brönsted Acid Sites of HSAPO-34			
	O(1)	O(2)	O(3)	O(4)
ΔE/(kcal/mol)	1.025	1.935	0	1.59
DPE/(kJ/mol)	1526	1522	1530	1524
proton charge (e)	0.677	0.655	0.669	0.66
R(O-H)(Å)	0.977	0.979	0.981	0.978
R(Si-O(H))	1.766	1.754	1.774	1.751
ν(O-H)/cm^−1^	3706	3684	3625	3700
ν(O-H)/cm^−1^ [3,56]	3625	3601	-	3630

## Data Availability

Data are contained within the article and Supplementary Materials.

## Supplementary Materials

The following supporting information can be downloaded at: https://www.mdpi.com/article/10.3390/molecules28248087/s1.
