# Supplementary material for: Simulating Crystal Structure, Acidity, Proton Distribution, and IR Spectra of Acid Zeolite HSAPO-34: A High Accuracy Study
Source: Molecules. 2023 Dec 14;28(24):8087. doi: 10.3390/molecules28248087 (PMC10745790; doi:10.3390/molecules28248087)
Supplement: Supplementary file 1 [file molecules-28-08087-s001.zip › molecules-2731852-supplementary.pdf]

Supplementary Materials for

Simulating Crystal Structure, Acidity, Proton Distribution, and IR Spectra of Acid Zeolite HSAPO-34: A High Accuracy Study

Xiaofang Chen * and Tie Yu

Institute of Molecular Sciences and Engineering, Institute of Frontier and Interdisciplinary Science, Shandong University, Qingdao 266237, China; yutie@sdu.edu.cn

* Correspondence: xf.chen@sdu.edu.cn or chen_smiling@163.com

Crystal structures for four possible Brönsted acid sites of HSAPO-34

1. HSAPO-34-O(1)

```
P    Si    Al    O    H
5    1    6    24    1
9.4007863147795252    0.0508416894817605    0.0211904411912341
-0.7173926710983457    9.4582456695708057    0.0686982704937082
-0.6841026581713295    -0.7863327170157070    9.2055510326969472

Direct
0.1042511338412808    0.3478431075150255    0.8773520667177692
0.3282944347212506    0.8838405176218700    0.1024105820101866
0.8905252618781461    0.6772356390838397    0.1071141163671854
0.1219879155513155    0.9064577958754825    0.6642047604566415
0.6623833510865751    0.1432792859340566    0.8780359462377731
0.8756929536718729    0.1212113290400936    0.3209284664855545
0.6586422300187493    0.9103201973954711    0.1146341650721823
0.9028366401723318    0.1318001627130485    0.6751351154089207
0.1215887738802053    0.6737233841664647    0.8825255451871570
0.3288527168953479    0.1084304805086234    0.8654822698932847
0.0873941814480261    0.8874384052349242    0.3102631089081740
0.8666487826210982    0.3522957760209309    0.0904012581481126
0.2318079041143548    0.2562633570908730    0.8825567017315947
0.9860650659773285    0.0201413017641627    0.2505487359470848
0.8571688072440296    0.2630168285588752    0.2431814218630493
0.3241828001447971    0.0221682270429824    0.0259287833665525
0.2584614501852514    0.9019849594547651    0.2461152414065495
0.0375756572043926    0.3510954263702288    0.0243593027269213
0.7659908335298411    0.7728981031384023    0.1038249947361152
0.9989868271220601    0.9871756732374593    0.7234415322682253
```

0.7318507038923840	0.1061094828904814	0.7369212801214076
0.9585719716823391	0.6822811720333348	0.9610130582019991
0.1166167559650475	0.7585682731126298	0.7215895581975289
0.6762844652302320	0.0188875787786458	0.9752758621089797
0.2559203672164685	0.7587072398467001	0.0044311016486915
0.9916575583718554	0.2889343070166674	0.7556732317789283
0.7112356080929843	0.0191448162650758	0.2816871319258709
0.7361623893766662	0.2796300376646812	0.9553582742649809
0.0014596120232397	0.7254920060689187	0.2344228567816060
0.2655716611470496	0.9902108371118175	0.7166001973413145
0.1088872256420075	0.8892837430691074	0.4986678440541539
0.5047486667347343	0.1646776721240855	0.8403860465298294
0.8283980044478909	0.5277365859673750	0.1274144903130789
0.8838799770618166	0.1400423883149600	0.4924963720665261
0.4844847781903283	0.8520277073280980	0.1393635685822758
0.1587075826015578	0.4980767550881566	0.8456968894899219
0.6456746964917457	0.0253898038441065	0.3586817289478432

2. HSAPO-34-O(2)

P	Si	Al	O	H
5	1	6	24	1

9.4157308065911103	0.0031910354574918	-0.0067964537344146
-0.7667122932949254	9.3553994270995684	-0.0016412245330089
-0.7087620886028244	-0.8459087228804921	9.3599126953852991

Direct

0.1102106266691933	0.3509961232949692	0.9051036155752357
0.3346665683509187	0.8952815442259876	0.1382785258235728
0.8912482094525132	0.6891909240620819	0.1470282705444959
0.1129090469586060	0.9072709192253043	0.6939406710970388
0.6570867322867358	0.1386343802594752	0.9162195934676447
0.8691254282658250	0.1260082006944643	0.3589120091048983
0.6603670283531216	0.9235915710806069	0.1440748306097888
0.8994751610480094	0.1426926890319820	0.6960911254502022
0.1170873387687692	0.6858504320450578	0.9211720103950682
0.3300158951864844	0.1187648780133230	0.9080263689155430
0.1007998970745376	0.8979376034463584	0.3541644356524856
0.8634666563548876	0.3576618970290539	0.1190657455988600
0.2453693785865951	0.2755619835255771	0.9363628476133457
0.0063020063992028	0.0433708288034111	0.3239688618649197
0.8989855923009742	0.2946304744422932	0.2938746035998747
0.3356611432571768	0.0378056687205728	0.0680870383151841

0.2645130431269394	0.9122897826926177	0.2790423644252797
0.0170055206549193	0.3388293543322547	0.0322628952136270
0.7644167845742729	0.7791829946467033	0.1460616922521396
0.9731854198997638	0.9785310560208984	0.7135075085037244
0.7480760804107049	0.1446980640236575	0.7898133819241764
0.9564402062762980	0.6920426879738599	0.0022875253775183
0.1021598596992419	0.7630425596766202	0.7598192010310143
0.6606037794002972	0.9911195774921495	0.9751893954615056
0.2538126164925103	0.7746373796442896	0.0362890213716724
0.0261553667218095	0.2825685537424647	0.7674587885858912
0.7239912869549769	0.0592006141042560	0.2720750924058990
0.7145549326514313	0.2587855550525120	0.0337988855580065
0.0035921158326886	0.7397260129733851	0.2717056798921291
0.2408596480944851	0.0051283590367603	0.7678361208844606
0.1355946779637094	0.8775728385234771	0.5357336823191190
0.5038353129878885	0.1654087115041776	0.8657691262635510
0.8352853556018971	0.5339358737737996	0.1676458122431512
0.8567722935514794	0.1747172754978052	0.5231035286833716
0.4873891652583282	0.8554607350614845	0.1701271067269303
0.1539633124897861	0.5083481522594937	0.8871251964260978
0.9222236063141338	0.3695788684478885	0.3727376861204590

3. HSAPO-34-O(3)

P	Si	Al	O	H
5	1	6	24	1

9.2026923298873484	-0.0437378805110530	-0.0604921434222307
-0.8806290466275346	9.4743797804724039	-0.0205687941270150
-0.6181758967282425	-0.6211151932364529	9.2765186868726843

Direct

0.1080905917148840	0.3364934904929555	0.9002222414935801
0.3178211757032230	0.8761567039724483	0.1125562994459841
0.8728810202711088	0.6718903451345994	0.1140634217881669
0.1071606054677616	0.8980063402666119	0.6806948564285591
0.6686371657924823	0.1320404392471914	0.9013621382580936
0.8709645921391598	0.1125173468089358	0.3405974963777822
0.6733672159172812	0.9089057076041200	0.1297705626728316
0.8930712514121808	0.1156534818921315	0.6877564001996817
0.1202744835419551	0.6628391322757139	0.9005692200284869
0.3136805637572095	0.0988426463692633	0.8905048921357022
0.0936112567242731	0.8797480707100149	0.3321897676991554
0.8618030134196601	0.3457595952092802	0.1068472735151573

0.2498578381548882	0.2639651186534735	0.9158052764843987
0.0356421735947379	0.0504920695882234	0.2866088242557510
0.8558441460558299	0.2502124426071575	0.2554039819468130
0.2564339366783059	0.0010668947896235	0.0359392012447088
0.2656935198612516	0.8801661090711193	0.2660168926484232
0.0393978059485391	0.3391329277556707	0.0466631760759952
0.7318290063775947	0.7427445565998596	0.0902923252535999
0.9669509659459408	0.9563011511591668	0.7288903114402032
0.7168824626236940	0.1101082896221399	0.7479648925806615
0.9528205684198525	0.6741749491931159	0.9746144590128978
0.1246559141773460	0.7561833479799489	0.7457540336198534
0.7283566759679374	0.0187623637021588	0.9943668320412868
0.2605975408454597	0.7353195310262990	0.0288861024651794
0.0006825168430638	0.2566605703257423	0.7839120321099884
0.7528288306441695	0.9793127632476839	0.2948679917481130
0.7287613802113881	0.2802744898605383	0.9677766200896798
0.9727773903346062	0.7493926858466153	0.2403350778218254
0.2409945255538020	0.0058580691300989	0.7285933272859211
0.0990982989805289	0.8723342556180711	0.5159910201851687
0.5015980389737322	0.1185988727983585	0.8934700292242184
0.8329661960786297	0.5202291263036471	0.1524034490145780
0.9012107419446167	0.1410699850302635	0.5097725318944626
0.4836186056038798	0.8925714723509159	0.1228923724112363
0.1474607519332807	0.4875815545709017	0.8569963153576055
0.0890048120668965	0.1057283062894783	0.2180504824915275

4. HSAPO-34-O(4)

P	Si	Al	O	H
5	1	6	24	1

9.1893104835269135	-0.1188333804240515	-0.1710558564472831
-0.8677941499145714	9.3388118195158007	-0.0912240327655137
-0.8396261447747343	-0.9354611198087019	9.3045287169213928

Direct

0.1499199527230033	0.3021980402897029	0.8671100197053008
0.3511043878535887	0.8455567997338065	0.0789186914316815
0.9105150576695209	0.6370887647091479	0.0790499180631130
0.1435233287305167	0.8504019966812209	0.6284063704294454
0.7068459126561564	0.0883399037263501	0.8709336455119754
0.9213070766873201	0.0793850424708751	0.3087814362370338
0.7071327710293005	0.8686865562274164	0.0963173757889351
0.9199684122671385	0.0706128339736551	0.6513137178504707

0.1427456691912283	0.6251424381795445	0.8661304297929036
0.3503497803801494	0.0648869209992924	0.8569697678547357
0.1438939003024728	0.8584807975050452	0.3076824785366412
0.9154091210958555	0.3128826617256664	0.0805822585129121
0.2965702043675194	0.2378274233873299	0.8844142739107923
0.0803120248010885	0.0249177538942646	0.3332488005501162
0.9120022725117209	0.2205998325315903	0.2284875862510631
0.2935379440007964	0.9650377204828615	0.9933349349580567
0.3111217380429636	0.8673615963005759	0.2332057346438745
0.0873421447626654	0.3181262967021681	0.0123983175027433
0.7623426261169612	0.6978656482339360	0.0580764786523318
0.9906397394733020	0.9077679606596689	0.6215918497047639
0.7501756316989230	0.0591280597303054	0.7169854257374197
0.9717267231543829	0.6205892767144405	0.9320056509193151
0.1422394766065267	0.7252798284453945	0.7205356026089405
0.7627755710270918	0.9723343479618194	0.9603104372298645
0.2815485319764619	0.6970074308204062	0.0035650843378292
0.0406944701361311	0.2012536033065473	0.7567125292538179
0.7892810418415834	0.9523612715464083	0.2609729879483922
0.7778407838688111	0.2388484584157382	0.9390137683546271
0.0198329353997053	0.7376568476279743	0.1884689760486680
0.2636541222856010	0.9740370488512298	0.6924709502711508
0.1723348119699040	0.7937699341459350	0.4767173613447113
0.5395499809262887	0.0818718035018975	0.8587834443560638
0.8843828188324494	0.4893897614054055	0.1343172546673941
0.8887146976173810	0.1457922059059484	0.4821178198886855
0.5173779197047139	0.8530519006921082	0.0831531650589952
0.1750197958330943	0.4490100050616377	0.8091983650405936
0.8542226261622545	0.2409750345355022	0.4833022318941644
